# A novel method for calculating the dynamic capillary force and correcting the pressure error in micro-tube experiment

**DOI:** 10.1038/s41598-017-16870-9

**Published:** 2017-11-29

**Authors:** Shuoliang Wang, Pengcheng Liu, Hui Zhao, Yuan Zhang

**Affiliations:** 1 School of Energy Resources, China University of Geosciences, 29 Xueyuan Road, Beijing, 100083 China; 2 Petroleum Engineering, Yangtze University, 1 Nanhuan Road, Jingzhou, 8060550 China; 3 Research Institute of Petroleum Exploration and Development, SINOPEC, 29 Xueyuan Road, Beijing, 100083 China

## Abstract

Micro-tube experiment has been implemented to understand the mechanisms of governing microcosmic fluid percolation and is extensively used in both fields of micro electromechanical engineering and petroleum engineering. The measured pressure difference across the microtube is not equal to the actual pressure difference across the microtube. Taking into account the additional pressure losses between the outlet of the micro tube and the outlet of the entire setup, we propose a new method for predicting the dynamic capillary pressure using the Level-set method. We first demonstrate it is a reliable method for describing microscopic flow by comparing the micro-model flow-test results against the predicted results using the Level-set method. In the proposed approach, Level-set method is applied to predict the pressure distribution along the microtube when the fluids flow along the microtube at a given flow rate; the microtube used in the calculation has the same size as the one used in the experiment. From the simulation results, the pressure difference across a curved interface (i.e., dynamic capillary pressure) can be directly obtained. We also show that dynamic capillary force should be properly evaluated in the micro-tube experiment in order to obtain the actual pressure difference across the microtube.

## Introduction

With the advancement in the extraction technologies, more oil/gas is now being produced from low and ultra-low permeability oil/gas fields over the world^1,2^. Because the pore radius of low and ultra-low permeability reservoir is in the micro/nanometric scale, the classical percolation model is unable to describe the fluid flow characteristics in tight oil/gas reservoir^3,4^. The micro-tube experiment is widely used to study the law governing microcosmic fluid percolation in the microtube^5–8^. The key parameters to be measured in the micro-tube experiment are pressure and flow velocity. The pressure data are measured by a manometer that is mounted on the connecting tube. One end of the microtube is connected with a large radius connecting tube, while the other end of microtube is connected with a flow velocity measurement tube. In the process of micro-tube experiment, the measured flow velocity is relatively accurate. But, the measured pressure is not easy to obtain because the pressure difference across the microtube is too small. At present, most researchers believe that the pressure difference between the ends of microtube equals to the manometer pressure minus the atmospheric pressure^9,10^. However, the current method used to figure out the actual pressure difference across the microtube is not accurate; the pressure difference between the two ends of microtube is not equal to manometer pressure minus the atmospheric pressure^10^. The pressure drop occurring across the microtube is mainly composed of three parts: (1) Pressure loss along the flow path due to fluid wall friction; (2) pressure loss due to the sudden change in the pipe diameter; (3) pressure difference across the fluid interface due to capillary pressure. Previous research found that the pressure loss along the flow path due to fluid-fall friction and pressure loss due to the sudden change in the pipe diameter can be neglected as they are much smaller than the pressure loss caused by the capillary^10^. It is reported that the pressure loss caused by the capillary pressure can reach about 4.0% when the interfacial tension is 71.92 mN/m and the radius of flow-velocity measurement tube is 320 micrometer)^10^. And with the increasing of the flow-velocity measurement tube diameter, the pressure loss caused by the capillary pressure tends to be increased. Therefore, the pressure loss caused by the capillary force cannot be ignored and the measured pressure difference across the microtube should be corrected by taking into account the capillary pressure effect. The pressure correction method is required for knowing the capillary force. Among the current pressure correction methods, the capillary force is static capillary force. However, during the process of micro-tube experiment, the gas/liquid interface is moving continuously along the flow velocity measurement tube. The capillary force in the flow velocity measurement tube should be the dynamic capillary force. There is a big difference between the dynamic capillary force value and the static capillary force value^11^. If the pressure is corrected by the static capillary force, not the dynamic capillary force, the pressure accuracy of the experiment will be affected.

The calculation method of dynamic capillary force is less discussed in the field of petroleum development, and discussed more in the field of hydraulic dynamics^12–14^. The static capillary pressure p_c_^stat^ is only a function of fluid saturation. The dynamic capillary pressure p_c_^dyn^ is a function of fluid saturation and fluid velocity^15^. Kalaydjian derived that dynamic capillary pressure minus static capillary pressure equals to $\tau \frac{\partial (\varphi {S}_{nw})}{\partial t}$, (*τ* is dynamic capillary force coefficient, dimensionless; *ϕ* is porosity, *f*; *S*_nw_ is non wetting saturation, *f*; *t* is time, s.)^16^. Hassanizadeh and Gray generalized dynamic capillary as ${P}_{c}^{stat}+\tau \frac{{D}^{s}(\varphi {S}_{nw})}{Dt}$, (*D* ^s^ is pore size in standard conditions)^17^. But, it should be noted that the concept of fluid saturation is difficult to be applied in the micro-tube experiment. As such, the dynamic capillary-pressure calculation method in the field of hydrodynamic theory cannot be used in the micro-tube experiment^18^.

This paper established a new method for calculating the dynamic capillary force and calibrating the pressure difference across the microtube in the micro-tube experiment. The glass plate experimental results and Level-set calculation numerical results are compared in this article for the first time. In the proposed approach, Level-set method is applied to predict the pressure distribution along the microtube when the fluids flow along the microtube at a given flow rate; the micro-tube used in the calculation has the same size as the one used in the experiment. From the simulation results, the pressure difference across a curved interface (i.e., dynamic capillary pressure) can be directly obtained. We also show in our study that dynamic capillary force should be properly evaluated in the micro-tube experiment in order to obtain the actual pressure difference across the microtube.

## Methodology

As mentioned above, the dynamic capillary calculation method applied to Hydrodynamic theory cannot be used in the micro-tube experiment. In this paper, a new method named as Level-set method was introduced to calculate the dynamic capillary pressure at pore throat scale. The Level-set method is proposed by Osher and Sethian^19,20^. Level-set method is mainly used in the field of intelligent control, image processing, etc^21^. Then, dynamic active contours method was introduced for segmenting objects by Kass^22^. In addition, the parametric active contours are represented explicitly as parameterized curves in a Lagrangian framework, and the geometric curves are represented implicitly as Level set of a two-dimensional function in an Eulerian framework. The basic idea is that the interfaces of two phases are represented as zero level set function^23^.

**Figure 1:**
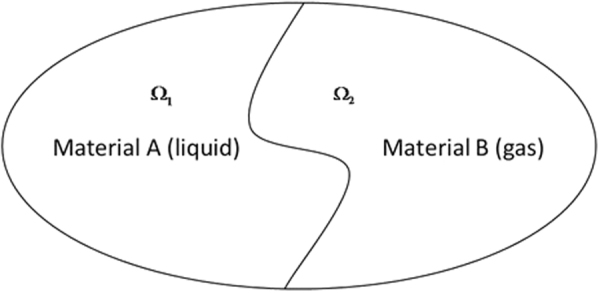
Schematic diagram of the interface between gas and liquid in the physical domain.

Figure 1 shows the schematic diagram of the interface between gas and liquid in the physical domain.

The Level-set method assumes that the function $\phi (\mathop{x}\limits^{\rightharpoonup },t)$ in flow field is moved at a certain speed. As shown in Fig. 1, the physical domain is ${\rm{\Omega }}={{\rm{\Omega }}}_{1}\cup \Gamma \cup {{\rm{\Omega }}}_{2}$. The subdomain Ω_1_ is material “A” and the subdomain Ω_2_is material “B”. In this paper, the material “A” represents the liquid, and the material “B” represents the gas. Gas and liquid are not immiscible.

In the domain of interest, the two-phase interface can be described as Γ(*t*). A new function $\phi (\bar{x},t)$ that satisfies Lipschitz continuity conditions is constructed. And the new function $\phi (\mathop{x}\limits^{\rightharpoonup },t)$ meets the following conditions^19,20^:

1 $$\phi (\mathop{x}\limits^{\rightharpoonup },t)=\{\begin{array}{c} > 0\quad \mathop{x}\limits^{\longrightarrow}\in {{\rm{\Omega }}}_{1}\\ =0\quad \mathop{x}\limits^{\longrightarrow}\in {\rm{\Gamma }}({t})\\  < 0\quad \mathop{x}\limits^{\longrightarrow}\in {{\rm{\Omega }}}_{2}\end{array}$$

Then, the position of the two-phase interface Γ(*t*) can be determined by the method of finding *φ* zero contour^19,20^:

2 $$\Gamma (t)=\{\mathop{x}\limits^{\longrightarrow}:\phi (\mathop{x}\limits^{\longrightarrow},t)=0\}$$

Solving the location of two phase interface is equivalent of solving the function *φ* or the next time step. The following equation describes how *φ* changes as time proceeds^19,20^:

3 $${\phi }_{t}+\vec{u}\cdot \nabla \phi =0$$

The velocity $\vec{u}$ is projected onto the normal direction of the two-phase interface. Let *x*(*t*) be the point track of the velocity of $\vec{u}$, we have^19,20^:

4 $$\frac{\partial \phi }{\partial t}+{F}_{N}(\mathop{x}\limits^{\longrightarrow},\nabla \phi ,k)|\nabla \phi |=0$$

The function *φ* in Eq. (4) is the distance function. *φ* being zero corresponds to the location of the interface. Since *F*_*N*_ is a function of $\nabla \phi$, we can use the Hamilton function $H(\overrightarrow{x},\nabla \phi )$ instead of the second term of Eq. (4). Eq. (4) can be simplified to the following Hamilton-Jacobi equation^19,20^:

5 $$\frac{\partial \phi }{\partial t}+H(\mathop{x}\limits^{\longrightarrow},\nabla \phi )=0$$

Eq. (5) is the Level-set equation. At present, the finite element method can solve the Level-set equations efficiently. After several years of research and applications, the accuracy of the Level-set solution method is verified^24–27^.

The Level-set method is a kind of implicit method which attempts to solve hyperbolic transport equation. The zero level set equation is considered as a function of the two-phase interface in a higher dimensional space, and the shape of the interface is obtained through solving Eq. (5) at each time step.

The Level-set method has been also successfully used to simulate two-phase fluid flow. The interface separates the two kinds of immiscible liquid and keeps moving as time elapses. In the process of fluid movement, the surface tension exists only at the moving interface. Using the Level-set method, we can transform the interface tracking problem into the problem of solving the zero-level set of the level set function. However, no previous research has examined the suitability and accuracy of Level-set method in studying the seepage law of oil and gas in microscopic pore spaces.

## Methods

### Verification of Level-set method

To verify the accuracy of Level-set method in studying the fluid seepage in microscopic pore spaces, we first compare the results of the interface-tracking experiments against those predicted by the Level-set method.

#### Experimental

**Figure 2:**
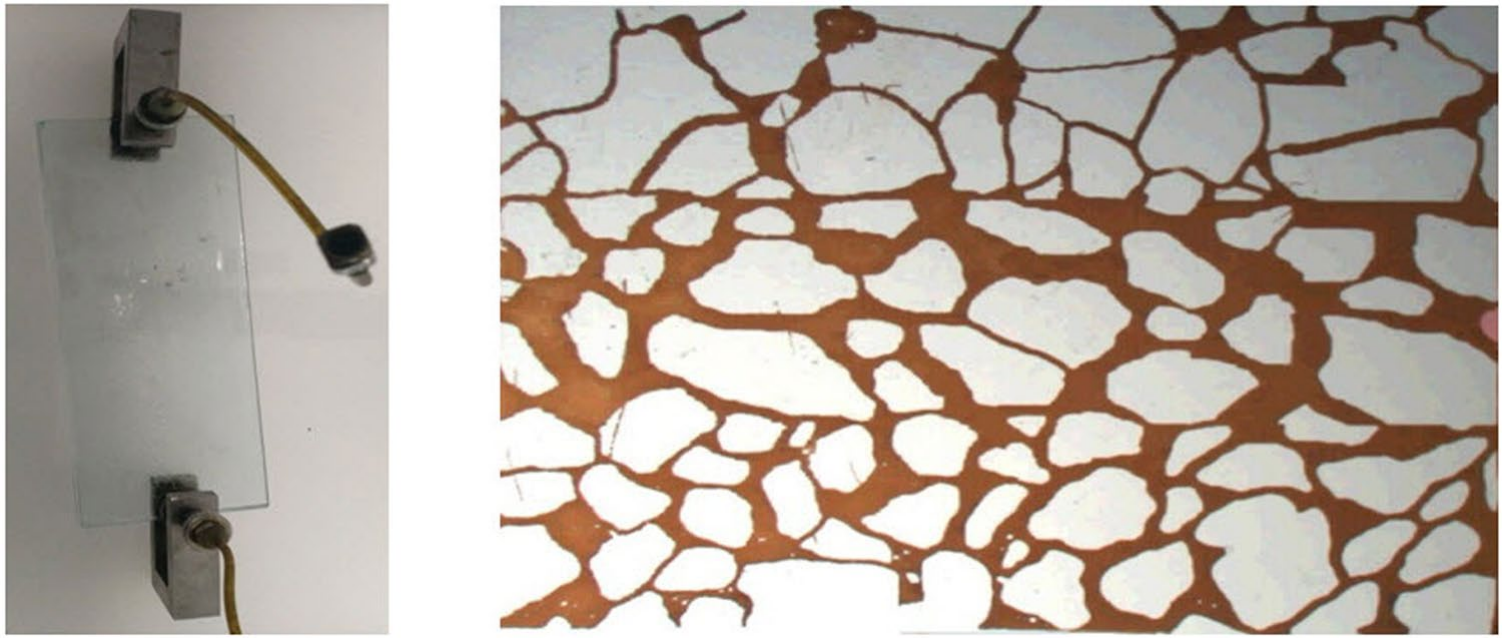
Top view of the micromodel (left) and magnified view of the pore throat structure embedded in the micromodel (right).

The interface-tracking experiments are conducted with a micromodel setup. The micromodel setup can be used to visually observe how the microscopic fluid seepages through a microscope. The pore throat structure, obtained from SEM images captured on the surface of real core samples, is carved on the micromodel. Figure 2 shows the general view and detailed view of the micromodel.

**Figure 3:**
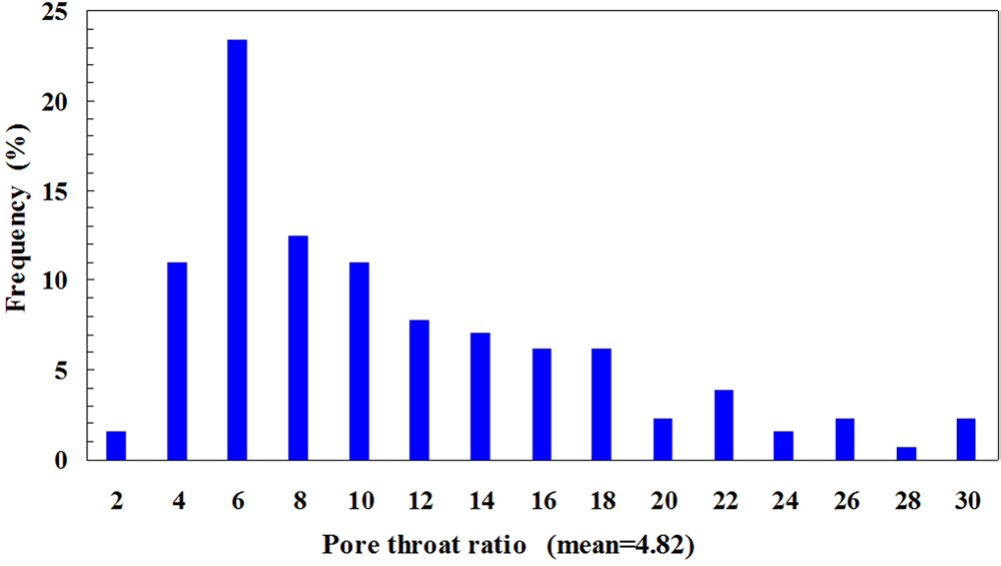
Pore/throat ratio distribution of the micromodel.

**Figure 4:**
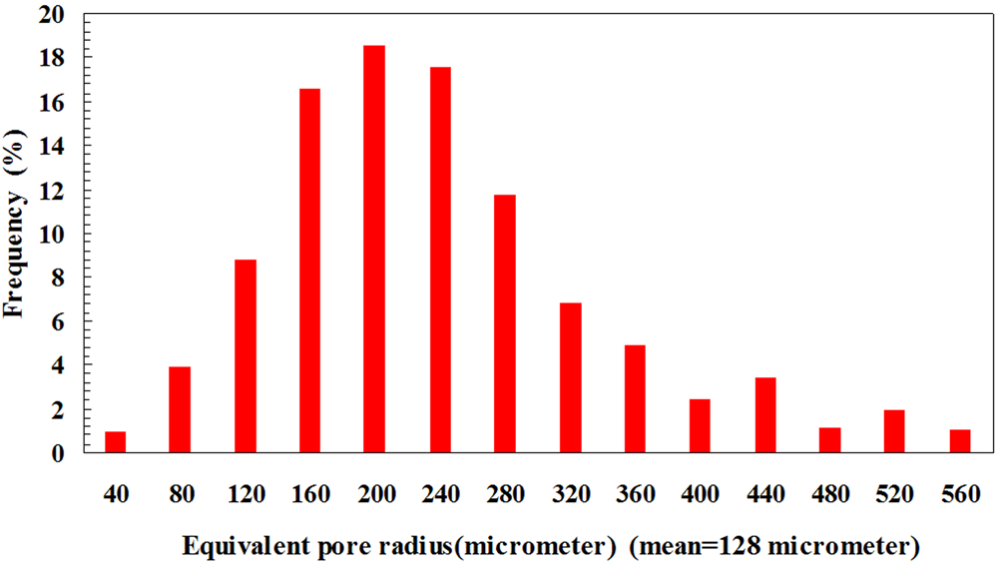
Distribution of the equivalent pore radius.

The pore/throat ratio distribution and pore radius distribution can be obtained by measuring the radii of the pores and throats in the micromodel. Figure 3 shows the pore/throat ratio distribution of the micromodel. The mean pore/throat ratio is found to be 4.82. Figure 4 shows the distribution of the equivalent pore radius; the equivalent pore radius is evaluated by considering the dimensions of all the pores and throats in the micromodel. The mean equivalent pore radius is 128 micrometer.

The oil used in the micromodel experiment is made of crude oil and kerosene. The viscosity of the oil is 2.0 mPa·s. The viscosity of water used is 1.0 mPa·s. The size of the glass plate model is 2.0 cm × 1.5 cm. A syringe pump is used to inject fluids into the micromodel. The fluid flow is observed by an optical microscope.

The detailed experimental procedures are as follows:

1. To saturate the micromodel, water was injected slowly into the glass plate model by the syringe.
2. Oil was then injected gently into the micromodel until no water production is observed. As such, the micromodel was saturated with connate water and oil.
3. The microscope was then turned on to monitor the fluid flow in the micromodel. Water was then injected through the inlet port of the micromodel. The whole procedure was also videotaped.
4. The microscope was used to observe distribution of oil and water in glass etching model. When the remaining oil in the pore was no longer changing, stopped the water flooding and end the video recording.

#### Theoretical methods for verifying the accuracy of Level-set model

The initial conditions of the Level-set numerical model were identical to those used in the micro-tube experiment. The detailed parameters are shown in Table 1.

**Table 1:** Initial parameters used in the Level-set model.

Parameters	Values	Parameters	Values
Water density (kg/m^3^)	1000	Water viscosity (mPa·s)	1
Oil density (kg/m^3^)	800	Oil viscosity (mPa·s)	20
Inlet pressure (kPa)	111	Interfacial tension (mN/m)	20
Outlet pressure (kPa)	101	Wetting angle (rad)	0.52
Mesh generation method	Free triangulation mesh	Mesh number	42648

**Figure 5:**
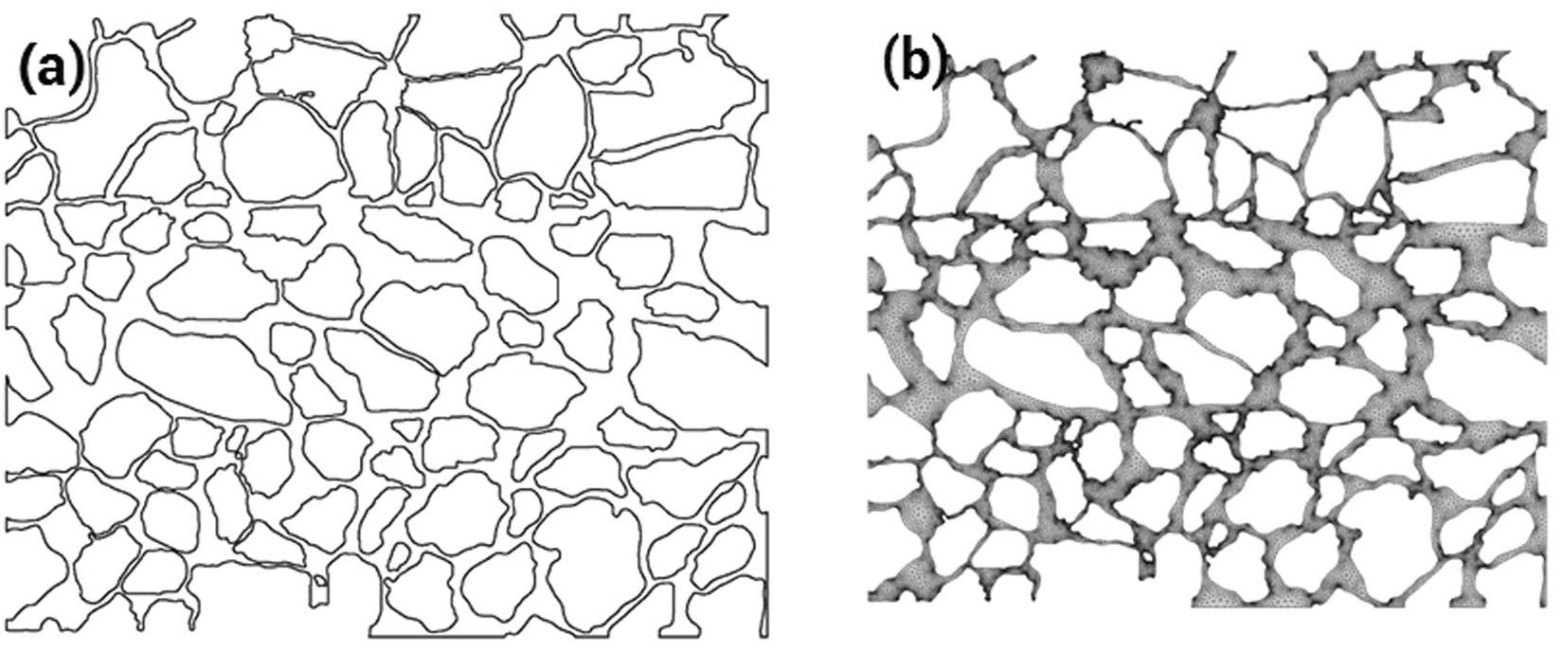
Pore structure diagram and mesh generation results. (**a**) The diagram of the pore structure obtained from the AUTOCAD software. (**b**) The results obtained from the mesh generation.

To compare the experimental results of the glass plate with the prediction from the Level-set numerical simulation, the pore-throat structure of the glass-plate etching model was imported into the AUTOCAD software. The image of the pore structure was draw with the spline curve and then zoomed to the true scale. Consequently, a mesh generation of the pore structure was made, and the grid system for numerical calculation was obtained. Figure 5 presents the diagram of the pore structure and the results obtained from mesh generation.

### Micro-tube experiments and theoretical calculation method of dynamic capillary force

#### Micro-tube experiments

**Figure 6:**
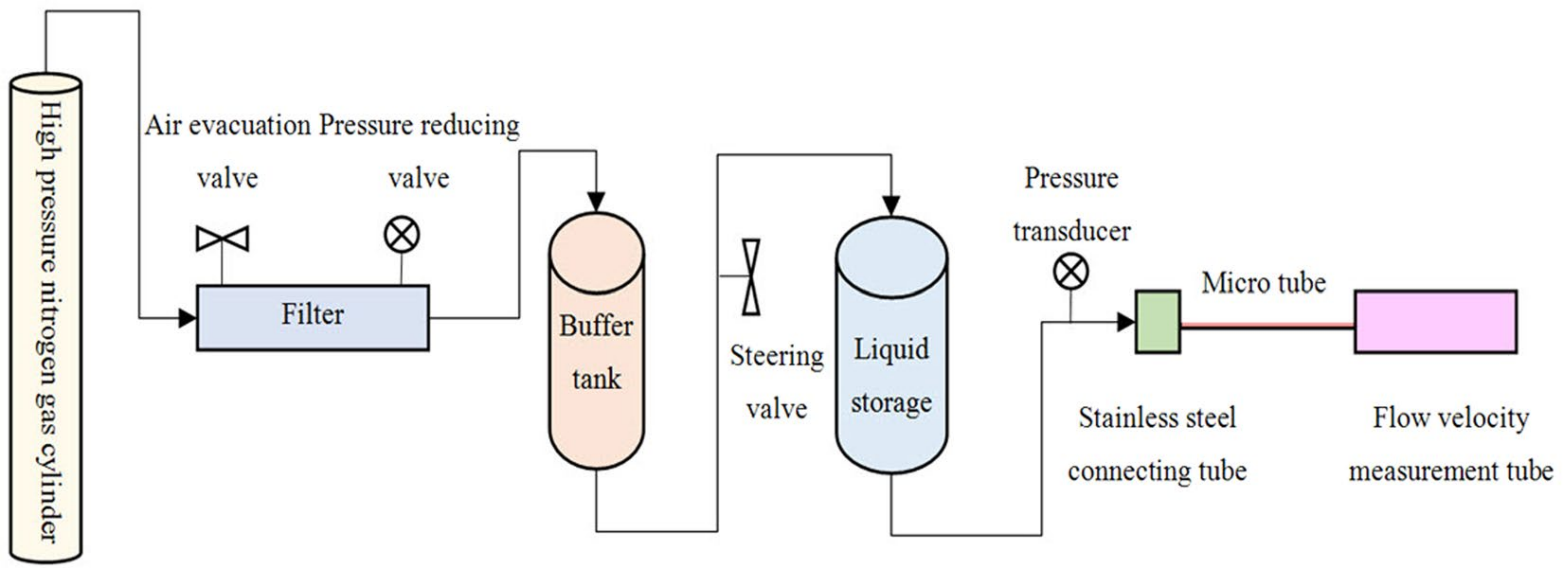
Flow chart of the micro-tube experiment.

**Figure 7:**
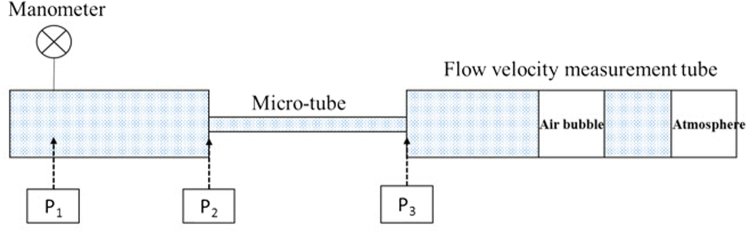
Schematic diagram of the micro-tube experiment.

Figure 6 shows the flow chart of the micro-tube experiment, while Fig. 7 presents the schematic diagram of the micro-tube experiment.

Three kinds of microtubes (Polymicro Technologies Company) were used in the micro-tube experiment; the internal radiuses of these microtubes were 10.0 micrometer, 15.0 micrometer, and 20.0 micrometer, respectively. The roughness of the microtubes was less than 5% determined by scanning electron microscopy (SEM). It can be considered that the inner wall is smooth.

The detailed experimental procedures are as follows:

1. The pipeline was firstly filled with nitrogen. The pressure in the pipeline was controlled at around 0.15 MPa.
2. The nitrogen was then transferred into the liquid storage tank through filter and buffer tank.
3. The liquid water in the liquid storage tank was transported to the microtube smoothly by nitrogen pressure.
4. The flow rate in the microtube was measured by the flow-velocity measuring tube.
5. Pressure was measured three times at a stable flow rate to confirm the reproducibility.
6. The system pressure was increased and the Procedure (5) was repeated.

#### Theoretical calculation of the dynamic capillary force

**Figure 8:**
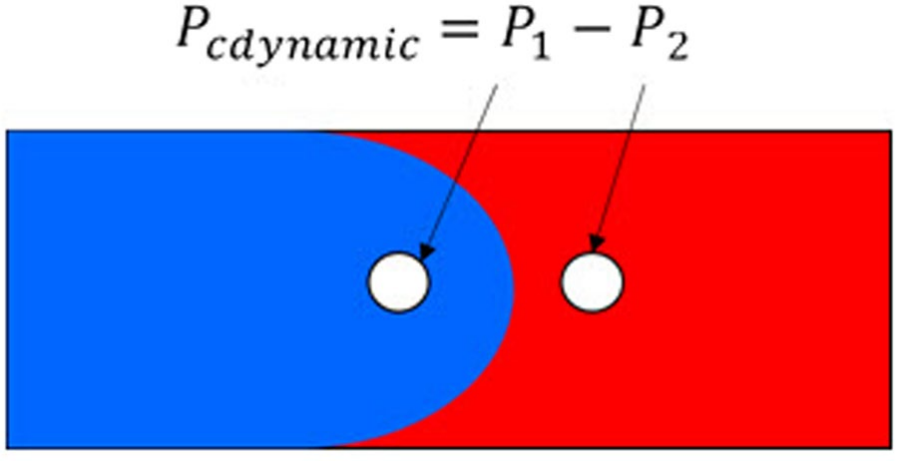
Schematic diagram of the dynamic capillary force.

Theoretically, the dynamic capillary force can be obtained by calculating the pressure on both sides of the contact surface. As shown in the Fig. 8, the blue region represents water, while the red region represents oil. P_1_ and P_2_ indicate the pressure on both sides of the two-phase interface, kPa. The value of the dynamic capillary force is defined as the pressure difference between P_1_ and P_2_. The key to calculate the dynamic capillary force is to obtain the accurate morphology of the oil and gas interface.

The Level-set method can be employed to calculate the dynamic capillary force in the micro-tube experiment. The detailed calculating steps are as follows:

1. By knowing the size of micro-tube experimental device, the geometric structure of the Level-set numerical simulation model was determined from the flow-velocity measuring tube. Grid subdivision of the model has been done which should satisfy the computational stability and description accuracy requirements.
2. Different material properties of the Level-set model were identified and assigned according to the micro-tube experimental fluid properties, i.e., fluid viscosity, interfacial tension and wetting angle, *etc*.
3. The boundary conditions and time steps of the Level-set model were loaded according to the micro-tube experimental process. For example: the velocity of the inlet and outlet of the experimental device.
4. Next, the Level-set model numerical simulation was carried out. The pressure value of P_1_ and P_2_ were recorded during the entire experimental process. Then, the dynamic capillary pressure was obtained by subtracting P_2_ from P_1_.
5. The results of the dynamic capillary force are used to correct the micro-tube experimental pressure.

**Figure 9:**
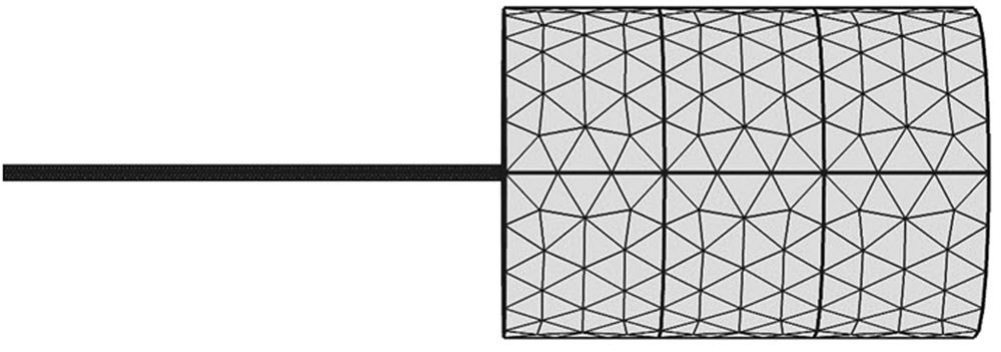
Mesh subdivision results from the numerical simulation model.

The specific parameters of the numerical simulation model were consistent with the experimental device. Grid subdivision for the model had been done which should satisfy the computational stability and description accuracy requirements. The mesh results of the numerical simulation were shown in Fig. 9.

The boundary condition of inlet was a constant pressure boundary, which was fixed at 0.15 MPa. The outlet of this model was connected with the atmosphere, and the boundary condition of outlet was also a constant pressure boundary, with a constant pressure of 0.1 MPa. According to the fluid distribution in the flow-velocity measuring tube, a curvature between the oil and gas phases can be observed in the numerical simulation model. The front part of the phase interface was oil, while the latter part of the phase interface was gas phase. The detailed parameters used in this model are shown in Table 2.

**Table 2:** Initial parameters used in the numerical simulation model.

Parameters	Values	Parameters	Values
Radius of the microtube (micrometer)	10, 15, 20	Wetting angle (rad)	1.40
Radius of the flow measurement tube (micrometer)	300	Interfacial tension (mN/m)	71.92
Length of the microtube (cm)	1.0	Inlet pressure (MPa)	0.15
Water density (g/ml)	0.99568	Outlet pressure (MPa)	0.10
Water viscosity (mPa·s)	0.8665	Temperature (K)	293
Mesh generation method	Free triangulation mesh	Mesh number	8394

The micro-tube experiment is mainly used to study the non-Darcy flow in micro/nano scale. Previously, it is believed that when pore radius is less than 20 micrometer, the fluid flow does not abide by the Darcy’s law and the Naiver-Stokes equation^28,29^. The objective of this numerical simulation model was to calculate the dynamic capillary force in the flow-velocity measuring tube. The radius of flow-velocity measuring tube was generally 300 micrometer. Therefore, the fluid flow in the low-velocity measuring tube was in accordance with Darcy’s law, which could be described by the Naiver-Stokes equation. The Level-set method could be used to calculate the dynamic capillary force in the flow velocity measurement tube.

Because the dynamic capillary force exits only in the flow velocity measurement tube, the numerical simulation model was established according to the scale of the flow velocity measurement in the physical simulation (Fig. 5).

## Results and Discussion

### Reliability verification of Level-set method

**Figure 10:**
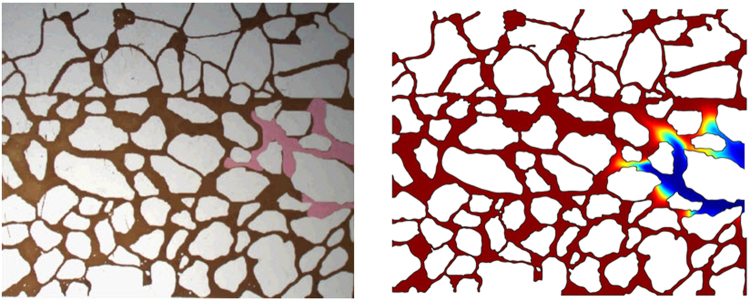
Experimental results (left) and the numerical calculation results (right) of the fifth time step.

**Figure 11:**
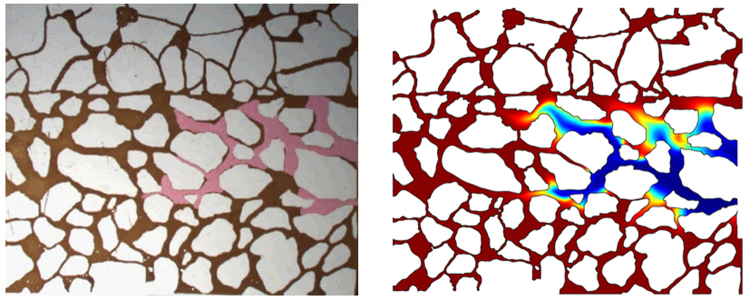
Experimental results (left) and the numerical calculation results (right) of the tenth time step.

In order to verify the accuracy of the Level-set algorithm, we compared the differences of the Level-set numerical results and the glass plate etching model experimental results. Figure 10 shows the experimental results (left) and the numerical calculation results (right) of the fifth-time step. As shown in Fig. 10(a), the pink region presents the water, and the brown region presents the oil. In Fig. 10(b), the blue region presents the water, while the red region exhibits the oil. Figure 11 shows the experimental results (left) and the numerical calculation results (right) of the tenth-time step.

Both experimental method and numerical simulation method have their limitations. The nature of experimental medium used to make the glass plate etching model is simple. For this reason, the glass plate etching model fails to characterize the complex wetting conditions of actual reservoir. Due to the quality of the experimental material, it is difficult to ensure that the wetting angles of all the position are the same in the glass plate etching model. Moreover, the experimental pressure cannot exceed 1.0 MPa under the influence of the model closure. On the other hand, The Level set numerical simulation method has two limitations. First, the computational accuracy of the Level set method is limited by the grid resolution. Secondly, it does not obey the mass conservation law. The mass loss is also related to the grid resolution. If the mesh is sufficiently refined, the computation task will increase exponentially. The amount of computation reached the level that the existing computer cannot afford.

From Figs 10 and 11, it can be found that the experimental results and the calculation results are consistent in the speed and sweep situation. But there are some differences between the experimental results and the calculation results in some regions. The main reason causing such difference is that the wetting angel of the glass plate etching model is inconsistent with the Level-set model. In the Level-set model, the wetting angle is about 0.52 rad in all areas, while it is difficult to ensure that the wetting angles is 0.52 rad in all areas because of glass material inhomogeneity in the actual glass etching model.

Generally, the analysis shows that the Level-set calculation results are in good agreement with the experimental results of the glass plate etching model, suggesting that the Level-set method can be used to calculate the seepage law of oil and gas at the pore throat scale. Based on the above analysis and discussion, the Level-set method has proven to be a reliable calculation method for describing the morphology of oil and gas interface at pore throat scale.

### Dynamic capillary force calculation and the pressure error correction in the micro-tube experiment

**Figure 12:**
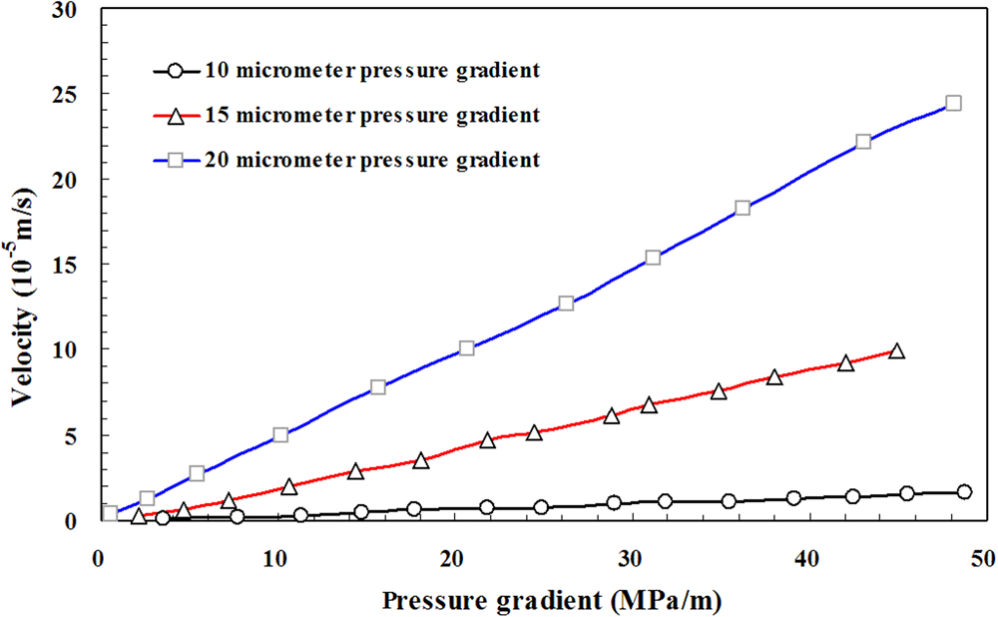
Experimental results of the micro-tube experiment.

Figure 12 shows the specific experimental results of the micro-tube experiment. The pressure gradient and flow velocity of different micro-tube diameters were obtained by using the micro-tube experiment. The pressure results are calculated by using the static capillary force in Fig. 12. As mentioned above, the pressure results in Fig. 12 should be corrected by using the dynamic capillary force.

**Figure 13:**
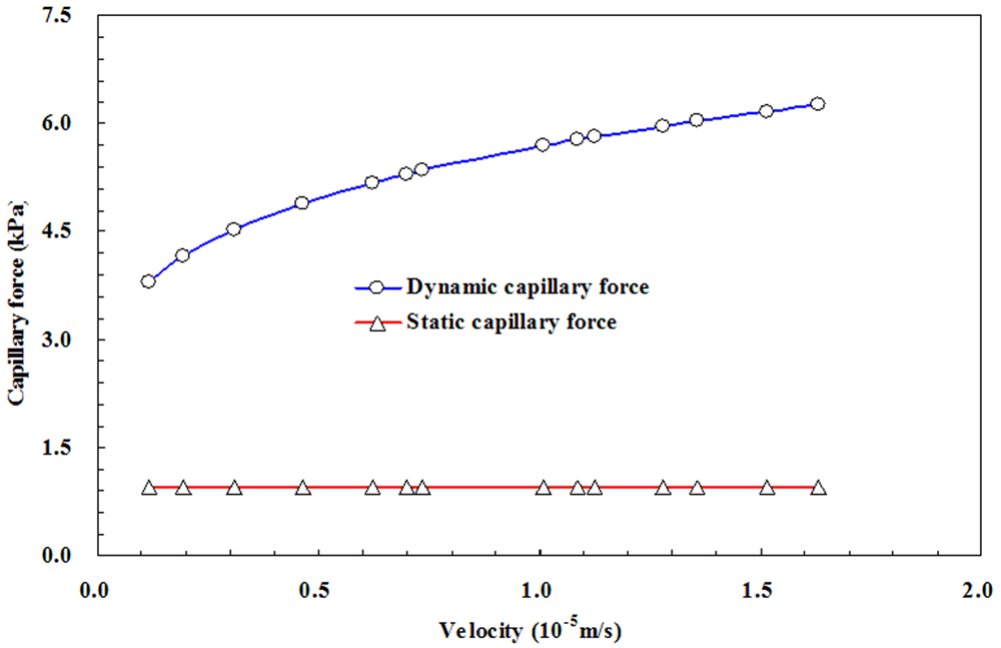
The calculated dynamic and static capillary forces.

In the section of the computational results of the experimental error correction, the parameters used in the numerical simulation model were consistent with the experimental apparatus. The dynamic capillary force of different micro-tube radius can be obtained by using the Level-set method. Figure 13 presents the dynamic capillary force and the results from the static capillary force calculation under the conditions of different displacement velocities in the flow-velocity measuring tube.

From Fig. 13, we observe that the dynamic capillary force is always higher than the static capillary force at the same velocity. The absolute difference between dynamic capillary force and static capillary force increases continuously as the flow velocity increases.

**Figure 14:**
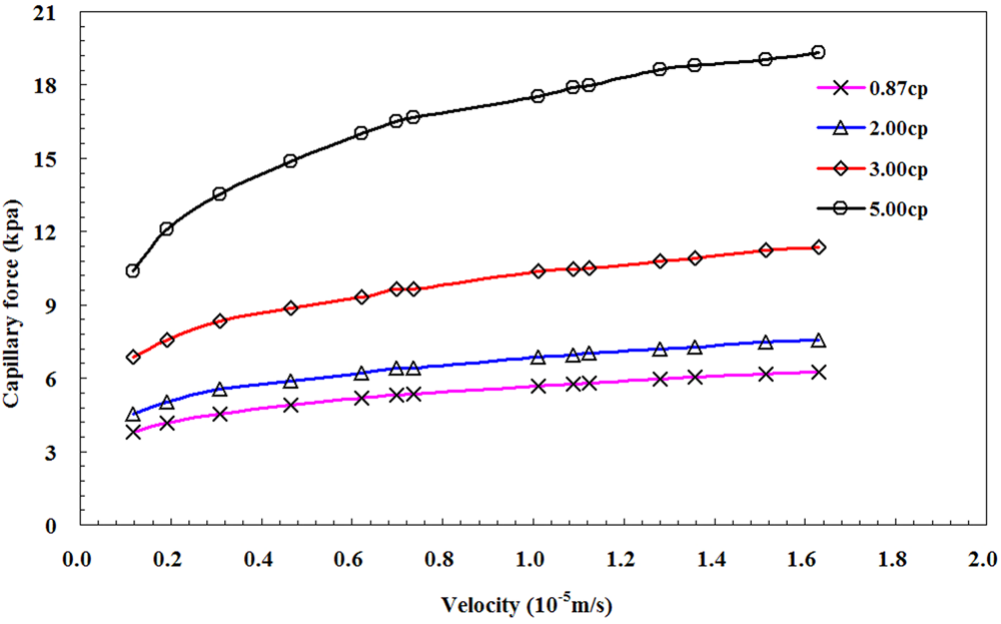
Dynamic capillary force in terms of viscosity for different oil samples.

As shown in Fig. 14, the dynamic capillary force changes with the viscosity of crude oil. The interfacial tension between oil and water phases is proportional to the viscosity of the crude oil. As a result, the numerical value of dynamic capillary force increases.

**Figure 15:**
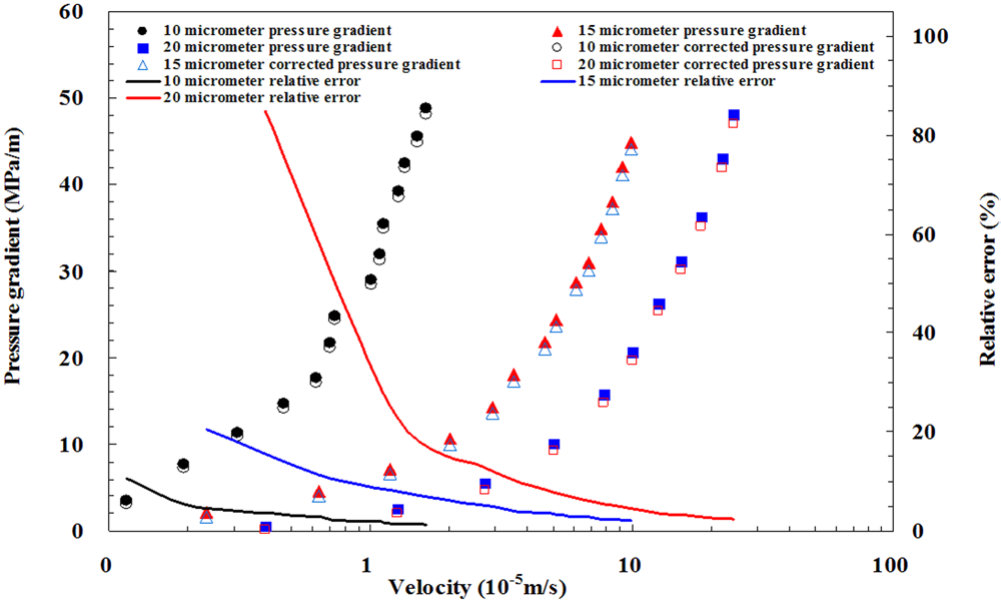
The corrected experimental pressure using dynamic capillary force.

Base on the measured dynamic capillary force, the actual experimental pressure can be corrected, as shown in Table 3. The corrected experimental pressure is shown in Fig. 15.

**Table 3:** Pressure error in the micro-tube experiments.

Radius of microtube (micrometer)	Velocity (10^−5^m/s)	Differential pressure between the ends of microtube (kPa)	Dynamic capillary force (kPa)	Corrected differential pressure between the ends of microtube (kPa)	Relative error (%)
10	0.12	35.44	3.80	31.65	10.71
	0.19	77.11	4.15	72.95	5.39
	0.31	113.18	4.53	108.64	4.01
	0.47	146.75	4.90	141.86	3.34
	0.62	176.60	5.18	171.42	2.93
	0.70	217.64	5.30	212.35	2.43
	0.74	248.74	5.35	243.39	2.15
	1.01	289.78	5.69	284.08	1.96
	1.09	319.00	5.78	313.23	1.81
	1.12	354.45	5.81	348.64	1.64
	1.28	391.76	5.97	385.79	1.52
	1.36	424.72	6.04	418.68	1.42
	1.51	455.19	6.17	449.02	1.36
	1.63	487.52	6.26	481.26	1.28
15	0.24	21.14	4.32	16.82	20.41
	0.63	46.00	5.19	40.81	11.29
	1.19	72.11	5.88	66.23	8.15
	2.02	106.29	6.54	99.76	6.15
	2.93	143.58	7.05	136.53	4.91
	3.52	180.26	7.32	172.94	4.06
	4.67	218.17	7.76	210.40	3.56
	5.15	244.27	7.92	236.35	3.24
	6.14	287.78	8.22	279.56	2.86
	6.81	309.53	8.40	301.13	2.71
	7.60	348.69	8.60	340.10	2.47
	8.40	380.39	8.78	371.61	2.31
	9.23	420.17	8.95	411.21	2.13
	9.94	449.38	9.10	440.28	2.02
20	0.40	5.59	4.75	0.83	85.06
	1.27	26.09	5.96	20.13	22.83
	2.74	54.66	6.95	47.70	12.72
	5.00	101.24	7.87	93.37	7.78
	7.78	157.14	8.64	148.51	5.50
	10.04	206.22	9.12	197.10	4.42
	12.62	262.75	9.57	253.18	3.64
	15.40	311.81	9.99	301.82	3.20
	18.29	362.73	10.36	352.37	2.86
	22.18	430.42	10.80	419.62	2.51
	24.41	481.36	11.03	470.33	2.29

The differential pressure between the ends of microtube is obtained by using static capillary, as shown in Table 3. The corrected differential pressure between the ends of the microtube is obtained by using dynamic capillary. Because the size of the flow-velocity measuring tube remains unchanged, the value of dynamic capillary force is only related to the flow velocity of the experimental fluid. The greater is the flow velocity of the experimental fluid, the greater is the corresponding differential pressure, and the greater is the value of dynamic capillary force. With the increase of the fluid velocity, the change rate of dynamic capillary force is less than that of the differential pressure at both ends of the microtube. Therefore, the faster the experimental fluid flow velocity, the smaller the differential pressure error will be. However, the maximum differential relative error can reach as high as 85% when the flow velocity is about 0.4 × 10^−5^ m/s. Therefore, it is necessary to correct the experimental pressure using the dynamic capillary force.

As depicted in Fig. 15, the variation trend of the corrected experimental pressure is almost consistent with the experimental pressure before the correction, while the numerical value has obvious deviation from the experimental results. If the differential pressure error correction is not performed by using dynamic capillary force, the inevitable error cannot be ignored.

## Conclusions

1. We analyzed the reasons that may cause the pressure error in micro-tube experiment, and figured out the shortcomings of the current pressure error correction method. The dynamic capillary pressure should be determined and used to calculate the pressure of the microtube rather than the static capillary pressure.
2. The glass plate experiment was used to verify the accuracy of the Level-set method in studying the seepage law of oil and gas at the pore-throat scale. According to the pore structure in the glass etching model, the Level-set numerical simulation model was established. By comparing the experimental results with the results obtained from the Level-set calculation, the Level-set method can be used to calculate the dynamic capillary pressure at the pore-throat scale.
3. We proposed a new method for calculating differential pressure using the Level-set method. According to the actual size and fluid properties of the micro-tube experimental model, a Level-set numerical simulation model was established. The dynamic capillary force corresponding to different flow velocity was obtained using the Level-set method. The absolute difference between dynamic capillary force and static capillary force increase continuously as the flow velocity increases. With the increase of viscosity of crude oil, the interfacial tension between oil and water is proportional with the viscosity of the crude oil, increasing the dynamic capillary force.
4. By analyzing the pressure between the ends of the microtube, the maximum differential relative error can reach as high as 85% when the flow velocity is about 0.4 × 10^−5^ m/s. Therefore, it is necessary to correct the experimental pressure using the measured dynamic capillary force. If the differential pressure error correction is not performed, the inevitable error cannot be ignored. It is pointed out that the micro-tube experimental pressure should be corrected by using the method proposed in this paper.
